# Consistent bacterial selection by date palm root system across heterogeneous desert oasis agroecosystems

**DOI:** 10.1038/s41598-019-40551-4

**Published:** 2019-03-11

**Authors:** Maria J. Mosqueira, Ramona Marasco, Marco Fusi, Grégoire Michoud, Giuseppe Merlino, Ameur Cherif, Daniele Daffonchio

**Affiliations:** 1 King Abdullah University of Science and Technology (KAUST), Biological and Environmental Sciences and Engineering Division (BESE), Thuwal, 23955-6900 Saudi Arabia; 2 University of Manouba, ISBST, BVBGR-LR11ES31, Biotechpole Sidi Thabet, Ariana, 2020 Tunisia

## Abstract

Highly productive conventional agroecosystems are spatially embedded in resource-homogeneous systems and count on generally nutrient-rich soils. On the contrary, desert oases are isolated, the soil is relatively poor, but yet productivity is similar to conventional agroecosystems. Soil dominates over plant as the main factor shaping root-associated microbiomes in conventional agroecosystems. We hypothesize that in desert oasis, the environmental discontinuity, the resource paucity and limited microbial diversity of the soil make the plant a prevailing factor. We have examined the bacterial communities in the root system of date palm (*Phoenix dactylifera*), the iconic keystone species of the oases, grown in heterogeneous soils across a broad geographic range (22,200 km^2^ surface area) of the Sahara Desert in Tunisia. We showed that, regardless of the edaphic conditions and geographic location, the plant invariably selects similar *Gammaproteobacteria*- and *Alphaproteobacteria*-dominated bacterial communities. The phylogeny, networking properties and predicted functionalities of the bacterial communities indicate that these two phyla are performing the ecological services of biopromotion and biofertilization. We conclude that in a desert agroecosystem, regardless of the soil microbial diversity baseline, the plant, rather than soil type, is responsible of the bacterial community assembly in its root systems, reversing the pattern observed in conventional agroecosystem.

## Introduction

Plants and their associated microbiota are not standalone entities, but a collective and unique ecological unit known as a holobiont^1^. The microbiota genome, which carries plant growth promoting (PGP) traits^2^, and the host genome act synergistically to favor the fitness and phenotypic plasticity of the holobiont^3–5^. Plants attract and sustain microbes mainly from the surrounding soil^6^ by depositing carbon into the rhizosphere via their roots^7^. In a stepwise process, the root system recruits and enriches microorganisms into the rhizosphere and the root surface further selects those that enter the root endosphere^8–10^.

A variety of biotic and abiotic factors shape the microbial communities associated with root systems. These include plant species, soil type, biogeographical location, plant community diversity and agricultural practices^11–13^. Whereas it is well established that in conventional agroecosystems soil type and agricultural practices play a major role in shaping the root-associated microbiomes^11,12,14,15^, few studies are available for agroecosystems in arid regions (see for instance Köberl *et al*., 2011^13^), such as desert oases. These are productive agroecosystems that provide multiple ecological and socioeconomic services to its human inhabitants through the exploitation of water resources and application of desert farming techniques^16–19^.

In conventional agroecosystems plants are characterized by frequent crop turnover, where plant species are periodically rotated, and are cultivated in a continuum, i.e. they are surrounded by other crops or by other types of vegetation. Their soils are generally rich and harbor high microbial diversity levels^12,14^. On the contrary, desert oases are embedded in and surrounded by the large, resource-scarce and harsh desert, which has low soil phylogenetic and functional microbial diversity^20–24^, but they host a higher plant community diversity characterized by a simultaneous multi-cropping system^17^. Oases are highly productive rural systems typical of desert regions in North Africa and the Middle East that provide not only agricultural, but also important social services to their inhabitants^17,19,25^. Owing to the presence of date palms (*Phoenix dactylifera*), the environmental conditions inside the oasis are milder than those of the surrounding desert. Date palms provide shade, decrease air temperature and maintain relatively high air humidity inside the oasis, enabling agricultural production^17,26,27^. Due to their long life cycle (40–50 years of economic life) and long history of cultivation^19^, domesticated date palm trees are considered to have coevolved with the oasis and its agricultural practices, similarly to plants growing in natural (uncultivated) systems^11^.

Due to the oasis isolation and the resource scarcity of the desert soil, we hypothesize that plants (i.e., date palm) should prevail over the soil as selection factor. In specific, we hypothesize that i) desert oasis plants select a specific, conserved and coevolved taxonomic and functional PGP core microbiota in their root systems, and ii) this plant-directed selection^13^ prevails over those imposed by the soil and agricultural practices.

To test these hypotheses, we examined the bacterial community diversity and networking properties in both the bulk soil and the root system (i.e., the rhizosphere and root tissues) of date palms (*P*. *dactylifera* cultivar *Deglet Nour*) across a broad latitudinal-longitudinal transect (22,200 km^2^ surface area) encompassing seven oases with different environmental settings, including the sea coast, mountains, sand dunes and the saline soil regions along the northern edge of the Tunisian Sahara Desert.

## Results

### Variability of soil characteristics in traditional Tunisian oases

Traditional oases are spread across the central and southern regions of Tunisia. These oases are environmentally diverse, ranging from the Mediterranean coast to the West Aures Mountains and the Grand Erg Oriental in the Sahara Desert (Supplementary Fig. S1). Farmers in these oases follow traditional subsistence agricultural practices to produce dates from the *Deglet Nour* L. date palm tree. According to chemical and physical analyses (Supplementary Table S1), the seven oases selected for this study were characterized by significantly different soils (PERMANOVA: F_6,14_ = 4.7923, *p* = 0.001, multiple comparisons in Supplementary Table S2). At the same time, as expected, the date palm root system significantly modified the surrounding soil compared to the bulk soil at all the studied oases (PERMANOVA: F_1,14_ = 8.0514, *p* = 0.001).

### Ranking the drivers of date palm root bacterial community composition, structure and diversity in oasis ecosystems

**Figure 1:**
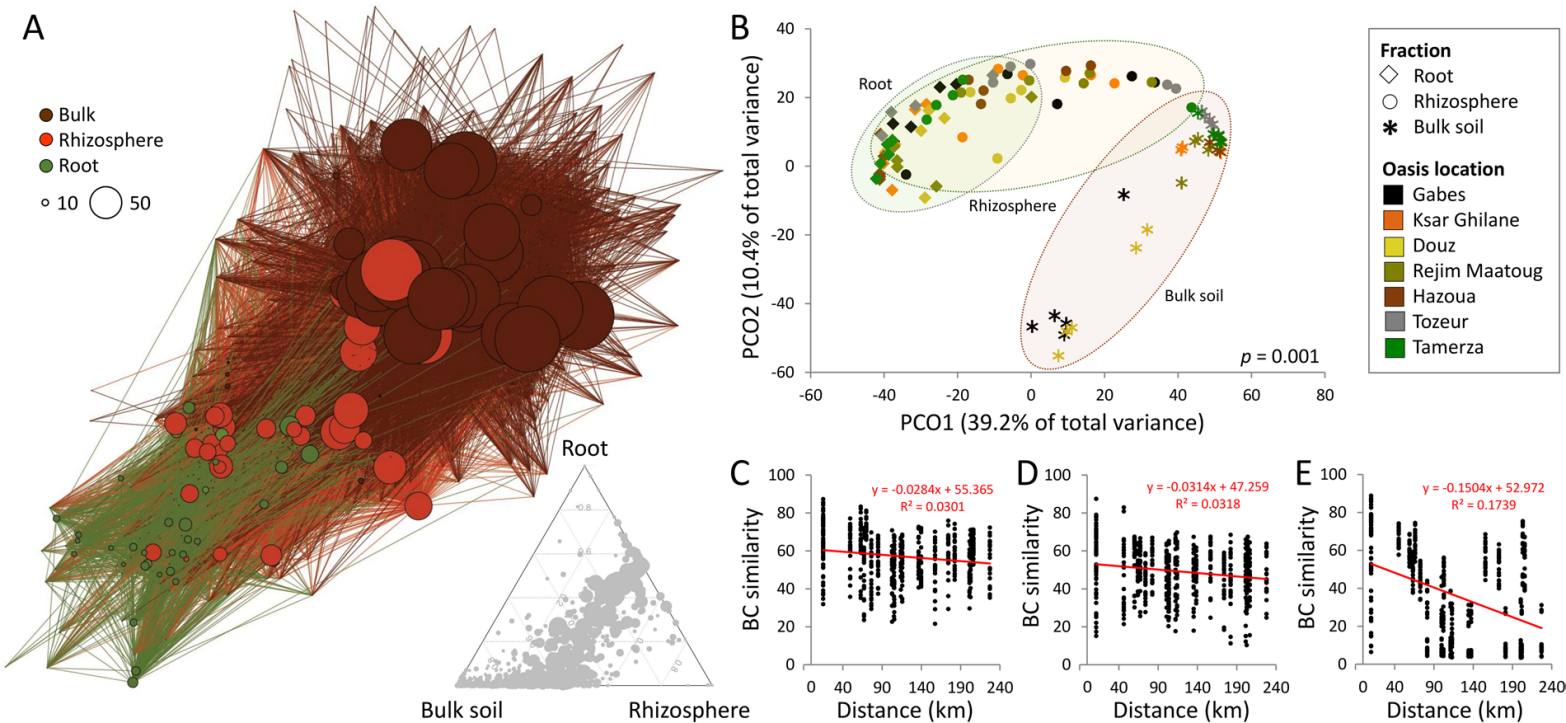
Structure and comparison of the microbial communities associated with the date palm root system at seven Tunisian oases. (**A**) Bipartite network analysis of bacterial communities associated with the date palm root system and bulk soils representing sample/OTU interaction. Sample nodes are depicted according to the environmental source and the plant compartment. OTU nodes are in grey, with edges connecting sample nodes to OTU nodes colored according to the compartments (i.e., fractions): dark brown = bulk soil; light brown = rhizosphere; green = root. Node size is proportional to the degree of connection. Ternary plots indicate the distribution of OTUs across the root, rhizosphere and bulk soil. The size and position of the gray circles indicate the relative abundance and affiliation, respectively, of the OTUs within the three fractions. (**B**) Principal Coordinate Analysis (PCoA) of bacterial communities from the date palm roots, rhizospheres and oasis bulk soils; the sample variation is given in Bray-Curtis distances. The symbols correspond to the ‘Fraction’, and the colors to the ‘Location’ of each oasis. (**C**–**E**) Distance-decay patterns (**C**, root; **D**, rhizosphere; **E**, bulk soil) as a linear regression between the Bray-Curtis similarity of the bacterial community and the linear geographic distances (km) among the oases. The relationship is tested by a linear correlation coefficient (R) with a significance (*p*) probability estimate.

After the removal of non-target DNA-sequences co-extracted from chloroplasts and mitochondria of plant, a total of 2,721,958 quality-filtered pair-end reads were retrieved from the 105 samples, representing the root system (root and rhizosphere) of *P*. *dactylifera* and corresponding bulk soils from the seven oases. The sequences were separated into 1,251 unique operational taxonomic units (OTUs, cut-off level, 97%). The highest number of overall unique OTUs was found in the bulk soils (1,233; 836 ± 321 per sample), followed by the rhizospheres (1,140; 753 ± 140 per sample) and the root tissues (624; 264 ± 65 per sample). The separation pattern of the bacterial communities in each root-system compartment consistently presented a spatial gradient from the bulk soil to the endosphere, passing across the rhizosphere (Fig. 1A and B). A group of 587 OTUs (47% of total OTUs) was shared among all three fractions (Fig. 1A), although subsets of specific OTUs were recruited only by the rhizosphere (535 OTUs) and endosphere (19 OTUs), suggesting that the date palm root system is a specialized niche for some taxa. Notably, the trend was observed at all the oases (Supplementary Table S3).

This selection process delineated specific bacterial microbiomes associated with the root and rhizosphere fractions. A principal coordinate analysis (PCoA) of the OTU composition revealed a ‘horseshoe-shaped’ ordination of the samples stratified from the root to the rhizosphere and bulk soil, explaining up to 49.6% of the dissimilarity in the community composition (Fig. 1B). A significant effect of the interaction between ‘Fraction’ and ‘Location’ was observed on betadiversity (GLM, df = 12,84 Dev = 25114, *p* = 0.001) explaining the 12% of the total deviance, followed by the individual factors ‘Location’ and ‘Fraction’, which explained 16% and 21%, respectively (Supplementary Table S4). A covariance analysis of the Bray-Curtis similarities in the bacterial community across 240 linear km, encompassing all seven oases, revealed a significantly larger decrease in the community similarity (ANCOVA, *p* < 0.0001) of the bulk soils (R^2^ = 0.17; 95% confidential interval = −0.1768 to −0.1240; n = 595; Fig. 1E) than in the roots (R^2^ = 0.03; 95% confidential interval = −0.04137 to −0.01541; n = 595; Fig. 1C) or rhizospheres (R^2^ = 0.03; 95% confidential interval = −0.04538 to −0.01746; n = 595; Fig. 1D), which only showed a slight decrease (~3%).

### Quantitative differences among the bacterial communities are explained by differential acquisition processes exerted by date palm root system fractions

**Figure 2:**
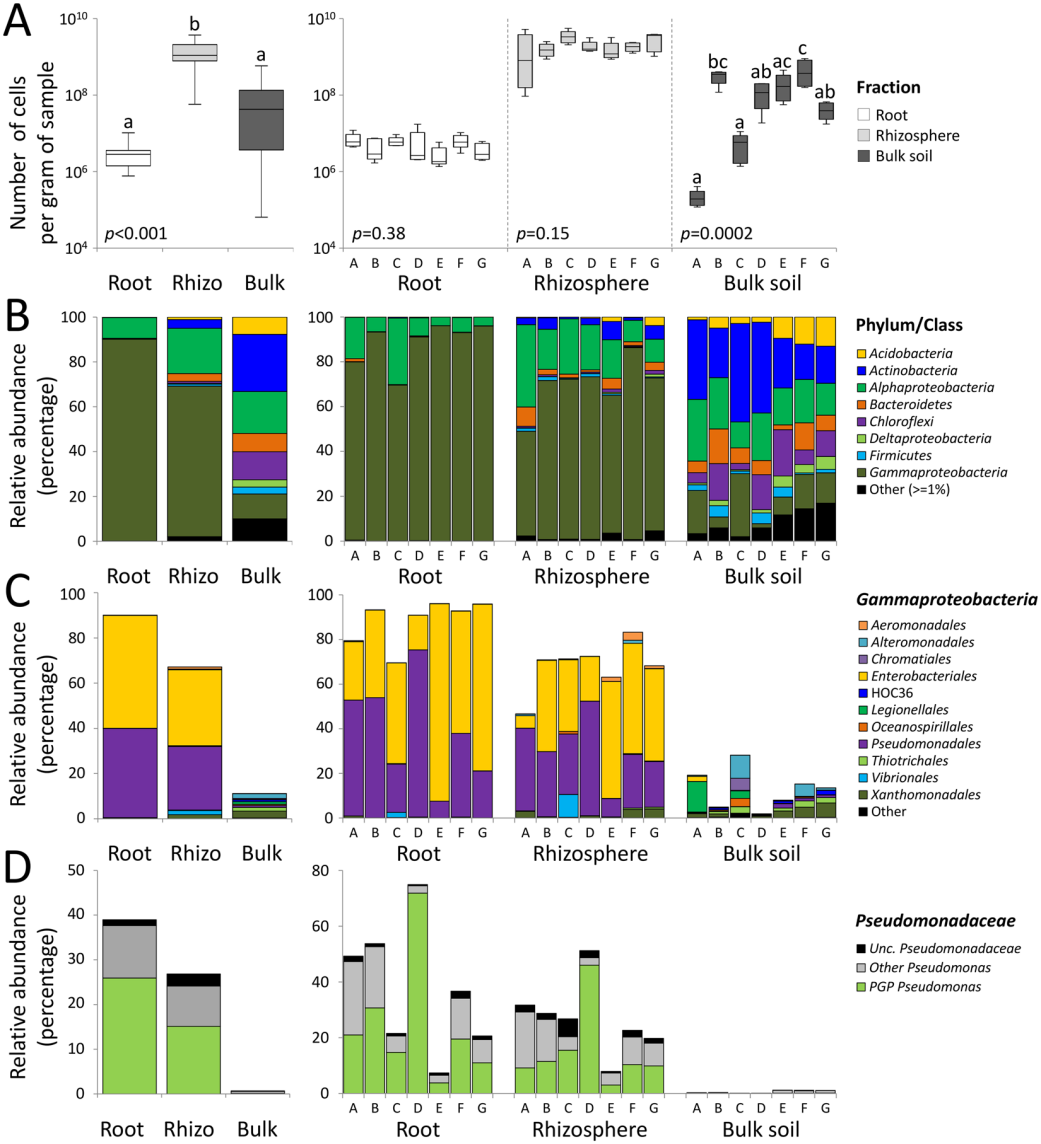
Average relative abundance of endophytic bacterial communities of the date palm root system and the oasis bulk soil at different taxonomic levels. (**A**) Quantification of bacterial cells colonizing the date palm root system (root and rhizosphere) and bulk soil across oases. Values are expressed as number of bacterial cells per gram of sample. Letters indicate statistical analysis (ANOVA) results from each fraction (left panel) and location inside the fraction (right panel). (**B**) Bacterial community compositions at the phylum/class level, including OTUs with more than 1% of the relative abundance reads. Taxonomic groups with less than 1% of the total reads are classified as “Other”. Relative abundance of OTUs belonging to the (**C**) *Gammaproteobacteria* orders and (**D**) *Pseudomonas* PGP taxa^28^ in date palm root systems across the different oases. The seven oases are reported with capital letters: A = Gabes, B = Ksar Ghilane, C = Douz, D = Rejim Maatoug, E = Hazoua, F = Tozeur and G = Tamerza.

Due to the different selective pressures of each fraction in the root system, their associated bacterial communities also hosted different bacterial numbers. Quantitative PCR showed that rhizosphere hosted the highest number of cells per gram of sample (1.9 ± 0.7 × 10^9^) compared to the root (4.5 ± 1.4 × 10^6^) and bulk soil (1.5 ± 1.6 × 10^8^) samples. The number of bacterial cells colonizing the root tissues and rhizosphere soils was similar across the oases (F_6,28_ = 1.11, *p* = 0.38 and F_6,28_ = 1.75, *p* = 0.15, respectively), whereas a significant effect of the oasis ‘Location’ was detected in the bulk soil (F_6,28_ = 6.82, *p* = 0.0002; Fig. 2A). Similarly, a progressive decrease in microbial alphadiversity (*richness* and *evenness*) was observed from the bulk soil to the rhizosphere and into the endosphere at all seven oases (Supplementary Table S5A). A multiple comparison test showed that the oasis ‘Location’ affected bacterial alphadiversity in the bulk soils only; no significant influence was detected in the bacterial communities of the date palm root system (root and rhizosphere; Supplementary Table S5B).

### Contribution of bacterial components to community diversity in the date palm root fractions

The taxonomic affiliations of the OTUs (Fig. 2B and Supplementary Table S6A) indicated that the date palm root system (root and rhizosphere) and bulk soil hosted 22 bacterial phyla, 62 classes (99.7% classified sequences), 102 orders (96%), 126 families (86%) and 122 genera (40%). While the bacterial communities were composed of the same dominant phyla/classes (i.e., *Alpha*, *Gamma* and *Deltaproteobacteria*, *Actinobacteria*, *Acidobacteria*, *Bacteroidetes*, *Firmicutes* and *Chloroflexi*), significant differences were found in their contributions to the different fractions (PERMANOVA: F_2,102_ = 81.15, *p* = 0.001; Supplementary Table S6A). The *Gammaproteobacteria* class dominated in the root endosphere (90%) and rhizosphere (67%) fractions at all the oases, but not in the bulk soils (11%). *Alphaproteobacteria* was the second most prevalent bacterial class in the rhizosphere and root compartments (20% and 9%, respectively), with a relatively large contribution to the bulk soil (19%). A decreasing contribution to the total bacteria community, from the bulk soils to the root tissues (bulk 54%, rhizosphere 9% and root 1%) of the pool including *Actinobacteria*, *Acidobacteria*, *Bacteroidetes* and *Chloroflexi* was observed at all the oases. The presence of some components, including *Firmicutes*, *Deltaproteobacteria* and others (contribution of less than 1% to the total community composition), was observed only in the bulk soils; there they reached up to 16%, with varying proportions at the different oases (Fig. 2B and Supplementary Table S6A). Notably, the oasis location only affected the taxonomic composition of the bulk soil (PERMANOVA: F_6,28_ = 5.4142, *p* = 0.001), and not that of the root-system bacterial communities (PERMANOVA, root: F_6,28_ = 1.1003, *p* = 0.053; rhizosphere: F_6,28_ = 0.7723, *p* = 0.066).

A peculiar taxa distribution of *Gammaproteobacteria*, the most abundant class, was observed among the fractions: the bulk soils hosted several orders (i.e., *Alteromonadales*, *Legionellales*, *Chromatiales* and *Xanthomonadales*), while the rhizosphere and root were always dominated by *Pseudomonadales* and *Enterobacteriales* (Fig. 2C). The *Pseudomonas* PGP endophytic bacteria E102 and E141, which were previously isolated from Tunisian date palm roots in desert oasis^28^, were also detected in our dataset, confirming that these two PGP strains are widely distributed in the root systems of date palms (Fig. 2D). The 16S rRNA sequences of E102 and E141 matched the *Pseudomonas*-related OTU_2 in the dataset (Supplementary Fig. S2). All the root tissues analyzed contained OTU_2, with an average relative abundance of 25 ± 8%. Moving away from the root, the relative abundance of OTU_2 decreased to 15 ± 5% in the rhizosphere, and less than 1% in the bulk soils. Interestingly (compared to the relative abundance of OTU_2 within the entire *Pseudomanas* community), OTU_2 represented the main component of this genus in the date palm root system (root = 63%, rhizosphere = 56% and bulk soil = 20%).

### Core bacterial components are highly conserved and networked in date palm root system communities across Tunisian oases

Despite the different environmental settings of the oases, the core bacterial microbiome of the date palm root-system fractions shared a considerable number of OTUs. A total of 73 (12% of root OTUs) and 309 OTUs (27% of rhizosphere OTUs) were always present in the date palm roots and rhizosphere, respectively. Although the shared OTUs were relatively limited in number, they represented 97% and 89% of the relative abundance of OTUs in the root and rhizosphere, respectively. In contrast, the core bacterial microbiome of the bulk soil included only 37% of the relative abundance of OTUs (145 OTUs, 12% of bulk OTUs). These core bacterial microbiomes reflected the taxonomic composition of the total communities as previously described. In the core microbiomes of the root and the rhizosphere, *Gammaproteobacteria* (94% and 71%, respectively) dominated the communities, with *Pseudomonadaceae* accounting for 48% and 43%, respectively (Supplementary Table S6B). Greater diversity was found in the core microbiome of the bulk soil; *Acidimicrobia*, *Actinobacteria*, *Alphaproteobacteria*, *Chloroflexi*, *Cytophagia*, *Acidobacteria* and *Bacilli* all together accounted for 76% of the overall core-composition (Supplementary Table S6B).

**Figure 3:**
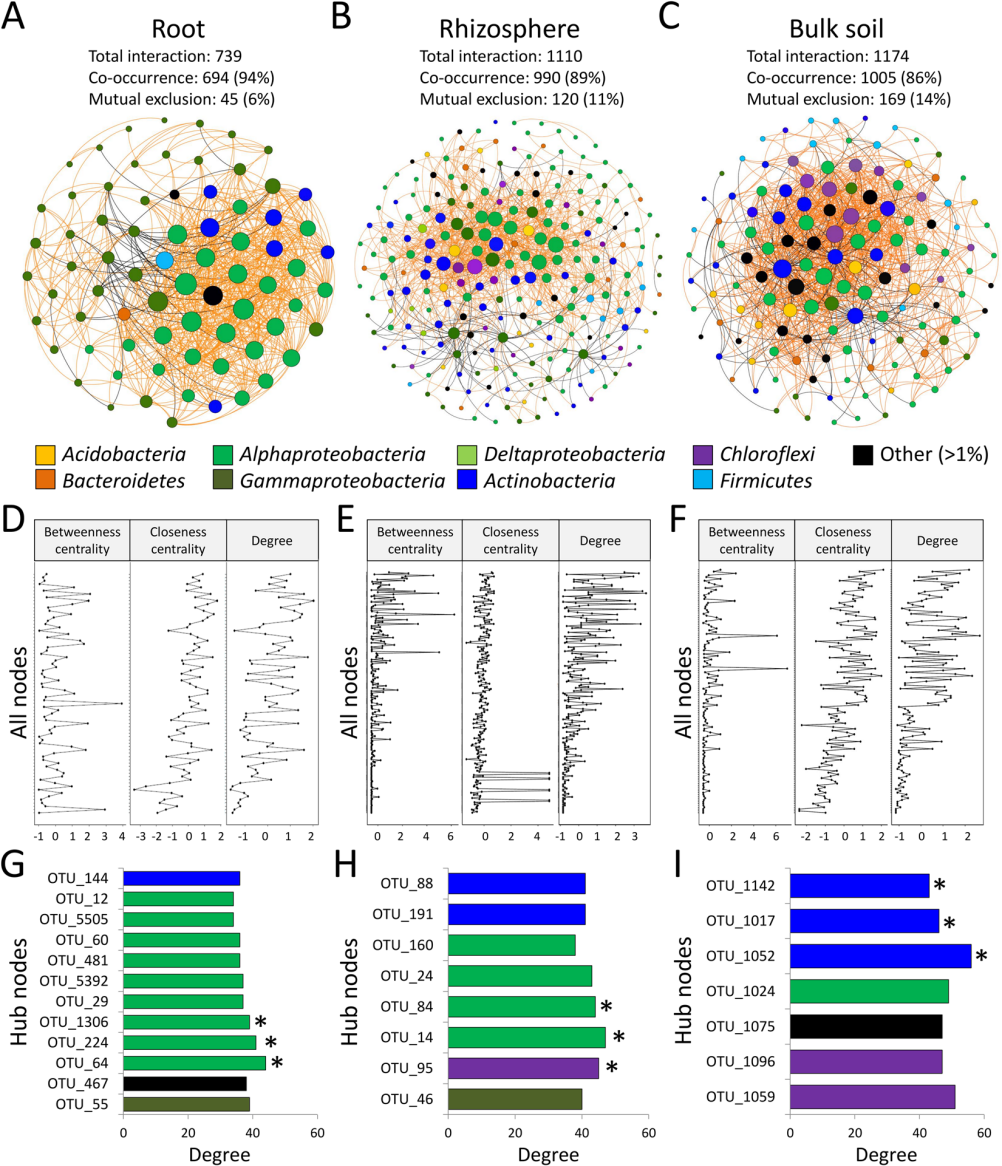
Co-occurrence and mutual-exclusion network analysis among core-bacteria components associated with the date palm root system fractions and bulk soil across oases. (**A**–**C**) Interaction among core microbial OTUs of the (**A**) root (n = 35), (**B**) rhizosphere (n = 35) and (**C**) bulk soil (n = 35). The nodes correspond to significant OTUs, and are colored according to their phylum/class affiliation (97%). The node sizes reflect their degree of connection (edge numbers assigned to the node). Edges (lines) connecting nodes indicate co-occurrence interactions (orange) and mutual exclusions (black). (**D**–**F**) The panel shows plots of two node centrality measures as well as the degree distribution for the (**D**) root, (**E**) rhizosphere and (**F**) bulk soil. (**G**–**I**) The nodes with more degrees are considered hub nodes typical of each fraction (G, root; H, rhizosphere; I, bulk soil). Star (*) indicates a keystone species (hub nodes with higher betweenness centrality).

The network co-occurrence analysis revealed that 100% of the OTUs in the core bacterial microbiome of the root, 74% in the rhizosphere and 93% in the bulk soil established significant ecological relationships. All fractions were characterized by unique network topologies (Table 1 and Fig. 3A–C). Significant higher numbers of co-occurrence interactions were recorded in the root (94%), and higher numbers of mutual exclusions occurred in the bulk soils (14%). Among all fractions, the root presented the highest clustering (0.58), density (0.28) and centralization (0.34) coefficients; the rhizosphere presented the lowest (Table 1).

**Table 1:** Topology indices of network interactions among significant bacterial components of the core communities in each fraction.

Topology Indices	Root	Rhizosphere	Bulk Soil
Number of nodes	73	230	134
Interaction	739	1110	1174
Co-occurrence	694	990	1005
Mutual exclusion	45	120	169
Degree	3032	4704	3746
Cluster coefficient	0.584	0.398	0.521
Centralization	0.339	0.165	0.294
Average path length	2.003	3.081	2.383
Average neighbors	20.247	9.652	17.522
Density	0.281	0.042	0.132
Heterogeneity	0.576	1.057	0.819

Fraction-specific variations in the taxonomic affiliation of the network nodes were detected (Supplementary Fig. S3A), as well as significant differences in the degrees of connection (GLM, Chi-square_2,434_ = 4184.9, *p* < 0.001; Figs 3D–F and S4). In the root network, the central interactions mainly occurred among *Gammaproteobacteria*, *Betaproteobacteria* and *Firmicutes*. Notably, in the networks of the bulk soil and rhizosphere where the taxonomic diversity of the nodes increased, a complex configuration of intra- and extra-phyla interactions was revealed (Supplementary Fig. S3B). In the bulk soil, *Verrucomicrobia*, *Bacteroidetes* and *Actinobacteria* led a high number of interactions; in the rhizosphere, the most central interactions were established by *Gammaproteobacteria*, *Bacteroidetes*, *Firmicutes* and *Planctomycetes* (Supplementary Fig. S3B).

In the bulk soil (Fig. 3I) and rhizosphere (Fig. 3H), 13 and 8 hub nodes, respectively, were represented by members of *Actinobacteria*, *Choroflexi*, *Alphaproteobacteria*, *Gammaproteobacteria* and others (i.e., *Betaproteobacteria*). In contrast, 12 hub nodes were detected in the root fraction (Fig. 3G), mainly belonging to *Alphaproteobacteria*. Among these hub nodes, keystone species (i.e., those having the highest betweenness centrality) were determined. In the root, three *Alphaproteobacteria* (*Rhizobium* and *Sphingopyxis*, Fig. 3G) were detected; in the rhizosphere, two *Alphaproteobacteria* (*Kaistobacter* and *Rhodoplanes*) and one *Chloroflexi* (Fig. 3H); and in the bulk soil, three *Actinobacteria* (*Acidimicrobiales*, Fig. 3I). *Pseudomonas* spp. OTU_2^28^ was not detected as a hub species, but it developed interactions with the bacterial components *Enterobacteriaceae* and *Sphingomonadaceae* in the endophytic community.

### Prediction of bacterial functional profiles in date palm root system

**Figure 4:**
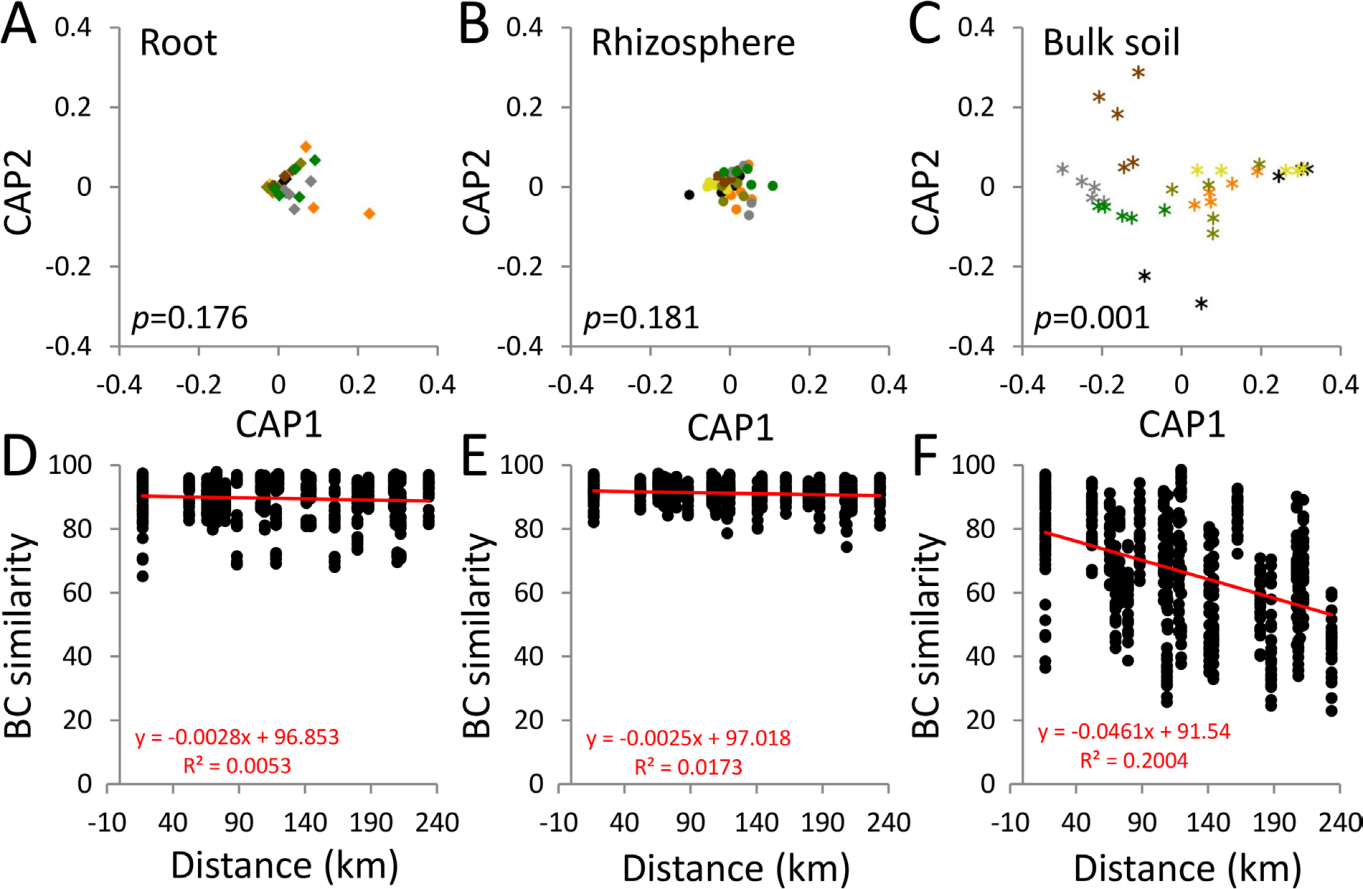
Functional predictions of each fraction’s core microbiota plant growth promoting (PGP) traits. Diversity of functional core microbiota in the three fractions (**A**, root; **B**, rhizosphere; **C**, bulk soil) is shown by constrained principal coordinate analysis (CAP). Distance decay patterns of date palm roots (**D**), rhizospheres (**E**) and bulk soils (**F**) across distance are calculated by linear regression using the functional Bray-Curtis similarities and linear geographical distances (km) among the oases. Color code from Fig. 1.

Canonical analysis of principal coordinates (CAP) showed that the PGP traits carried by the date palm bacterial microbiomes in the root (delta_1^2^ = 0.3206, *p* = 0.176) and the rhizosphere (delta_1^2^ = 0.4083, *p* = 0.181) fractions were not affected by the oases’ location (Fig. 4A and B, respectively). In the bulk soil, however, these functions were differently distributed across the different oasis soils (delta_1^2^ = 0.86515, *p* = 0.001; Fig. 4C). The linear regression between the microbiota functional similarities (Bray Curtis index) of each fraction and the linear geographic distances among the oases showed different distance-decay patterns (ANCOVA: *p* < 0.0001; Fig. 4D–F). In the bulk soil, the functional-microbiota similarity decreased with the distance between oases (R^2^ = 0.2004; 95% confidential interval −0.05353 to −0.03870; n = 595), whereas no changes were observed in the functional similarity along distance in the root (R^2^ = 0.0053; 95% confidential interval = −0.005834 to 0.0002933; n = 595) or the rhizosphere (R^2^ = 0.174; 95% confidential interval = −0.004097 to −0.001003; n = 595) compartments (Fig. 4D–F).

## Discussion

The root system of the date palm, analogously to what has been already demonstrated for many other perennial plants^8,29–31^, selects and enriches the bacterial diversity already present in the surrounding soil of the oasis. Through rhizodeposition, the date palm roots attract and enrich soil microorganisms into the rhizosphere, then the root tissues recruit them through a selective process mediated by the rhizoplane^8^. This recruitment process, in which a gradual differentiation in the bacterial community richness and composition occurs across the root system fractions^12,31^, was conserved among the different geographical settings studied in this work. As demonstrated by the high rate of distance decay, geographical location was a determinant of bacterial community selection only in the bulk soil. This can be ascribed mainly to the different geochemical settings of the oases, which were located across a territory of 22,200 km^2^. Soil-specific distribution of bacterial taxa (i.e. *Actinobacteria*, *Acidobacteria* and *Chloroflexi*^32–34^) could result from limited dispersal at different oases or local sorting mechanisms (e.g., agricultural management). Although agricultural practices homogenize soil, edaphic factors are still the main drivers of bulk soil bacterial diversity in oases^35^.

Despite the bulk soil hosting a higher bacterial biodiversity, the rhizosphere is colonized by more cells, owing to the carbon-rich environment created by date palm rhizodeposition^7^, which is constant and stable throughout the long life cycle of this perennial tree^25^. The bacterial communities in the root and rhizosphere are stabilized by the constant and strong selective pressure of the date palm root system, which has a strong protective and nutritional effect on the bacteria^7^. The selective enrichment triggered by plant root systems defines consistent core pools of bacteria typical of each fraction. In the date palm root system, *Gammaproteobacteria* is the dominant phylum enriched and selected from the microorganisms present in the oasis bulk soil^36,37^. The dominance and PGP benefits of *Gammaproteobacteria* in the bacterial community of root systems was already demonstrated for plants cultivated in other traditional desert agroecosystems^32,38–41^. In the date palm root system, *Gammaproteobacteria* is mainly represented by the order *Enterobacteriales*. This order includes many species found in enteric habitats. They originate from the traditional practices used to fertilize the soil, such as the application of natural fertilizers and cattle-assisted soil preparation, which can carry this kind of bacteria directly into the soil^42^. Besides *Enterobacteriales*, the *Pseudomonadales* group also plays an important role^28,40,43^. The presence of signatures from two *Pseudomonas* PGP strains (E102 and E141), which were previously described as bioprotectors of date palms under drought stress^28^, was revealed. Interestingly, the *Pseudomonas* PGP strains were associated with all the date palm roots analyzed here, representing 25 ± 8% of the total community in the root system. These data suggest a correlation between these PGP *Pseudomonas* strains and the date palm root system, supporting the hypothesis of functional cooperation with the plant host.

We judged the quality of our dataset as satisfactory, not only for the sequence coverage, but also in relation to the contamination by chloroplast and mitochondria 16S rRNA genes that were 31% and 1.5%, respectively, of the reads in the endophytic communities. Recently, alternative approaches have been developed for reducing the impact of non-target DNA on the sequencing process (PCR-clamps^44^, alternative primer sets^45^, treatment with restriction enzymes^46^). However, the effectiveness of such approaches largely depends on plant species and they do not exclude the introduction of new biases, including the lack of amplification of certain bacterial groups (i.e., abundant group in soil and a possible reduction of taxonomic coverage^47,48^.

The ecological importance of the fraction-core microbiota and potential interactions were also revealed by network analysis. The components of the root system’s core bacterial microbiome significantly interacted with each other, forming a complex ecological interaction web. The rhizosphere network was more complex than that of the bulk soil or root endosphere^49,50^. Bacterial taxa with high numbers of interactions (hubs) regulate other community members in the microbiome (either directly or indirectly) and maintain both the network structure and the ecosystem stability^51^. Most of the root network hubs were associated with *Alphaproteobacteria*, but *Gammaproteobacteria* also influenced the date palm root and rhizosphere by establishing interactions with other taxa. The structural roles of specific taxa (i.e., *Rhizobium* and *Sphingopyxis* in the root, and *Kaistobacter*, *Rhodoplanes* and *Chloroflexi* in the rhizosphere) were also identified, confirming that the stability of the fraction-specific microbiota is maintained by components that are specifically selected by the fractions^52^. Information about the ecological role of such bacteria in plant-microbe interactions is scarce, although several members of these genera have plant growth promotion and biofertilization properties (e.g., *Sphingopyxis*^53^), and also protect from biotic and abiotic stresses (e.g., *Rhizobium*^54^).

The date palm recruits a ‘functional core microbiota’ that performs essential services for the holobiont^3,40^, such as biopromoting (IAA production) and protecting services under abiotic stresses (ACCd production) such as drought^28^. Here, these earlier findings are validated, and a functional vicariance that is crucial for date palm tree resilience is guaranteed^30,55^. In the date palm root systems studied, the functional microbiota was conserved among the different oases, while the microbiota associated with the bulk soil was strongly influenced by the location of the oasis. This implies that the microbial PGP services are recruited similarly by the date palm root system across different environments. Indeed, in arid agricultural ecosystems similar to oases and subjected to desert farming practices, plant species-related factors were found to be important drivers in taxonomical and functional diversity^13,24^.

The date palm *P*. *dactylifera* originated in the area between the Nile and Euphrates rivers, and its cultivation was documented as early as 3700 BC^19^. It developed specific adaptations to the desert ecosystems of the Middle East and North Africa^56–58^. For example, the desert environment conditions can be extreme, with temperatures reaching 50 °C during the day. Under these conditions, water evaporation from the soil exceeds the plant capacity for water transport. The date palm can maintain high transpiration rates that are not compatible with a sole water supply from the soil. This transpiration is maintained by water reserves in the stem that are recharged during the night^56,57^. The adaptation of the date palm to the unique extreme conditions of the desert and its long history of cultivation in oases have led to its coevolution with the limited diversity of the desert microbiome. For example, earlier analyses of the genomes of endophytic *Enterobacteriales* members isolated from the date palm root tissues, such as *Enterobacter* strains (i.e., *E*. *asburiae* PDA134), confirmed that PGP traits that encourage salt tolerance *in planta* were mainly linked to hormone homeostasis^43^. At the same time, the order *Pseudomonadales* hosts several species, such as those isolated from date palm^28,59^, that have been used as biofertilizers, biostimulators and biocontrol agents in several plant systems^60,61^. This long coevolution has given the date palm the advantage of being able to select preferred beneficial microbial components.

Irrespective of the environmental setting of the oasis, the date palm always selects a conserved core microbiome that is able to deliver essential PGP traits. This difference from the microbial assembly strategy of crops in conventional agroecosystems has possible implications for plant fertility through manipulations of the root microbiome. PGP bacteria isolated from date palms in oases at a given location may be capable of colonizing date palms in other oasis ecosystems. Indeed, PGP pseudomonads isolated from the *Deglet Nour* date palm in a Tunisian oasis efficiently colonized Saudi Arabian cultivars^28^ that were phylogenetically divergent^19^. Therefore, future characterizations of the date palm core microbiota and its functionality in the oasis ecosystem will be instructive both in developing agricultural technologies that can improve crop production and sustainability in arid environments and, possibly, in restoring lands currently undergoing the desertification process.

## Methods

### Site description, sampling and processing

The area studied was approximately 22,200 km^2^ in Tunisia. Sampling was performed across a latitudinal-longitudinal range encompassing seven oasis ecosystems, including the Mediterranean coast of Gabes, the Grand Erg Oriental areas of Ksar Ghilane, Rjim Maatoug and Hazoua, the Chott salty-arid regions of Douz and Tozeur, and the Tamerza oasis in the Atlas Mountains, as shown in Supplementary Fig. S1. The selected oases have contrasting characteristics in terms of temperature, precipitation, geomorphological setting, traditional agricultural management and soil type (Supplementary Fig. S1, Supplementary Table S1). Sampling was conducted following the design described below at each site in November 2016. Five healthy date palm trees (*Phoenix dactylifera* cultivar *Deglet Nour*) of similar age were randomly selected from each of the oasis fields for the collection of root system samples (i.e., rhizospheric soil and root tissue). The sampling was authorized by the private owners at each location. The samples of the root system were collected using sterile tools at a depth of 30–60 cm around the trunk base, where the roots are more dense and active^62^. Processing to separate the rhizosphere soil (tightly attached to the root) from the root tissues was immediately performed^30^. Samples of the bulk soil (fractions of soil not influenced by the root system) were also collected from each location outside the oasis. All samples were stored at −20 °C.

### Soil chemistry

The chemical and physical properties of the root-surrounding soil and bulk soil were characterized at Geomar (Germany). Two replicates for each oasis and each soil type (root-surrounding and bulk soils) were analyzed for pH, conductivity (EC), total dissolved salts (TDS), organic matter (OM), total carbon (TC), total nitrogen (TN), TC/TN ratio and available element/nutrient (calcium, magnesium, potassium, ammonium, nitrate and phosphate).

### Total DNA extraction

Total DNA was extracted from the rhizosphere and bulk soil with the PowerSoil® DNA Isolation Kit (MoBio Inc., USA), starting from 0.5 g of each sample. For the root samples, total DNA was extracted from one gram of sterilized and grinded material^30^ using the DNeasy Plant Maxi Kit (Qiagen, Germany).

### Quantitative PCR (qPCR) of the bacterial community

Bacterial 16S rRNA gene copy absolute abundances were determined using the primer set Eub338/Eub518^63^. The qPCR reactions were carried out in triplicates for each sample on a Rotor-Gene Q thermocycler (Qiagen) with a reaction volume of 15 μl containing 1X GoTaq® Sybr Green Master Mix (Promega), 100 nM of each primer and 1.5 μl of template DNA. The qPCRs were run at 95 °C for 2’, 40 cycles at 95 °C for 15”, 53 °C for 20” and 60 °C for 20”; at the end of each run, melting curves of the PCR products were obtained through 91 cycles from 50 °C to 95 °C at a rate of 0.5 °C/cycle every 5 seconds. Standard curves were constructed from a series of dilutions ranging from 50 to 5 × 10^7^ copies/μl. R^2^ between 0.99509 and 0.99933 and amplification efficiencies between 93% and 96% were obtained. Conversion of the 16S rRNA gene copy to the bacterial cell number was calculated according to the average 16S rRNA gene copy number (GCN) of each sample obtained using Copyrighter^64^ and the results of the 16S rRNA gene Illumina MiSeq libraries.

### Sequencing and metaphylogenomic analysis of the 16S rRNA gene

Illumina tag sequencing of the V3-V4 hypervariable regions of the 16S rRNA gene was performed at KAUST (Bioscience Core Lab) using the primers 341 F and 785R^65^, according to procedures described by Mapelli *et al*.^49^. The raw sequence data were analyzed using a combination of QIIME (version 1.8) and UPARSE (version 8) pipelines^49^. A total of 2,721,958 high quality 16S rRNA gene sequence reads (average length, 314 bases) were obtained after quality filtering and paired-end merging of the 105 samples. These reads were clustered as OTUs, considering a 97% sequence distance similarity and taxonomy was assigned using QIIME’s UClust database. Chloroplast (27 OTUs, 2% of total OTUs) and mitochondria (3 OTUs, 0.2%) sequences were removed from the dataset. Chloroplast OTUs accounted for 31% of relative abundance in endophytic communities (root tissues) and for less than 0.05% in soil ones (rhizosphere and bulk soil), while mitochondria represented 1.5% of relative abundance in root and less than 0.0025% in soils. The Good’s coverage values, calculated after the removal of the chloroplast/mitochondria sequences, were higher than 0.96 for all the samples, allowing an adequate sequencing depth to study bacterial communities associated to date palm root system compartments (root, rhizosphere and bulk soil). The sequence reads were deposited in the NCBI SRA database under the BioProject ID number PRJNA417545.

### Bacterial diversity, taxonomy and statistical analyses

OTUs were filtered in order to keep only those presenting a relative abundance (%) higher than 0.005. A bipartite network analysis of the bacterial communities in the root tissues, rhizosphere and bulk soils was done with the QIIME script *make_bipartite_network*.*py* and visualized in Gephi^66^. For the principal coordinates analysis (PCoA), canonical analysis of principal coordinates (CAP) and multivariate analysis of deviance (multivariate generalized linear model, GLM), Bray-Curtis distance matrices were used. Two fixed and orthogonal explanatory variables were considered, ‘Fraction’ (3 levels: root/rhizosphere/bulk soil) and ‘Location’ (7 levels), as well as their interaction (‘Fraction’ × ‘Location’). Analysis of covariance (ANCOVA) was used to test whether the rate of community similarity decay (Bray-Curtis index) across the oasis locations was different among the three fractions (root, rhizosphere and bulk soil). Alpha diversity and ternary plots were calculated with PAST software^67^. The shared and unique OTUs for fractions and locations were represented in Venn diagrams (http://bioinformatics.psb.ugent.be). Statistical analyses to test differences in bacterial abundance and community composition among the fractions and locations were determined by ANOVA.

### Co-occurrence network analysis

The bacterial components (OTUs) composing the fraction-core microbiome (bulk soil, rhizosphere and root) were used with the CoNet plugin in Cytoscape 3.4 to perform a co-occurrence network analysis^68,69^. The network was built by combining the Pearson and Spearman correlation coefficients with the Bray-Curtis (BC) and Kullback-Leibler (KLD) dissimilarity indices. After 1,000 iterations of edge-specific permutations and bootstrap score distributions were performed to capture the similarity introduced by compositionality alone, the data were normalized to compute the statistical significance of the co-occurrence/mutual exclusion events. Then, the *p*-values were computed as indicated above by z-scoring the permuted null and bootstrap confidence intervals and using the pooled variance^70^. The clustering coefficient, the neighborhood connectivity distribution, the betweenness centrality and the topological coefficient were calculated as statistical descriptors of the networks^71^. For the visualization and characterization of the node centralization traits, the values of the betweenness centrality, closeness centrality and degree were normalized using a standardization method (n1). An analysis of deviance via GLM was performed using a quasi-Poisson distribution of error for the degree of connection, and a quasi-binomial distribution for the closeness and betweenness centrality. For the average path length, ANOVA was performed using a normal distribution. The obtained networks were visualized using Gephi 0.9.1^66^.

### Functional prediction

To predict the functional potential of the bacterial microbiota from the OTU tables, we used the Tax4Fun package^72^. It generates a relative abundance of the KEGG orthology (KO) groups for each sample based on matches between representative sequences from each OTU to organisms in the KEGG database. Raw sequence data were preprocessed for Tax4Fun with QIIME as described on the Tax4Fun website (http://tax4fun.gobics.de/). Representative sequences were selected and by using SILVA 132 taxonomic information was assigned by transforming the SILVA-based OTUs into a KEGG-organism taxonomic profile. The profile was normalized by the 16S rRNA copy numbers available from the NCBI genome annotations. The KO groups, relative to the PGP functions (auxin production, ACC deaminase activity, VOCs release, siderophore synthesis, nitrogen metabolism and phosphate solubilization), were extracted according to the KEGG database and the available literature^73–75^.

### Presence of *Pseudomonas* PGP strains E102 and E141 in Tunisian date palm root systems

The presence and abundance of reads related to two described PGP pseudomonads (*Pseudomonas* spp., strains E102 and E141), which were previously isolated from date palm root tissues^28^, were assessed and quantified. For this, the 16S rRNA gene sequences of the two isolates were blasted against the *Pseudomonas* OTU sequences in the dataset. The obtained sequences were aligned with Incremental Aligner SINA of SILVA^76^. Conserved-sequence blocks were identified with the Gbloks software^77^. The phylogeny of the identified OTUs (99%) was obtained with the Molecular Evolutionary Genetic Analysis MEGA7 by applying the neighbor-joining method with a bootstrap test using 1,000 replicates^78^.

## Supplementary information

Supplementary Information

Supplementary Table 1

Supplementary Table 6

## Data Availability

All data generated or analyzed during this study are included in this published article (and its Supplementary Information files).
