# Supplementary material for: Consistent bacterial selection by date palm root system across heterogeneous desert oasis agroecosystems
Source: Sci Rep. 2019 Mar 11;9:4033. doi: 10.1038/s41598-019-40551-4 (PMC6412053; doi:10.1038/s41598-019-40551-4)
Supplement: Supplementary file 1 — Supplementary Information [file 41598_2019_40551_MOESM1_ESM.pdf]

SUPPLEMENTARY INFORMATION for

Consistent bacterial selection by date palm root system across heterogeneous desert oasis agroecosystems

Maria J. Mosqueira[1]†, Ramona Marasco[1†]*, Marco Fusi[1], Grégoire Michoud[1], Giuseppe Merlino[1], Ameur Cherif[2], Daniele Daffonchio[1]*

[1]King Abdullah University of Science and Technology (KAUST), Biological and Environmental Sciences and Engineering Division (BESE), Thuwal, Saudi Arabia 23955-6900;

[2]University of Manouba, ISBST, BVBGR-LR11ES31, Biotechpole Sidi Thabet, Ariana, Tunisia 2020

* Correspondence to Daniele Daffonchio, daniele.daffonchio@kaust.edu.sa and Ramona Marasco, ramona.marasco@kaust.edu.sa

† These authors contributed equally to this work

SUPPORTING FIGURES

Figure S1. Picture, location and description of selected oasis.

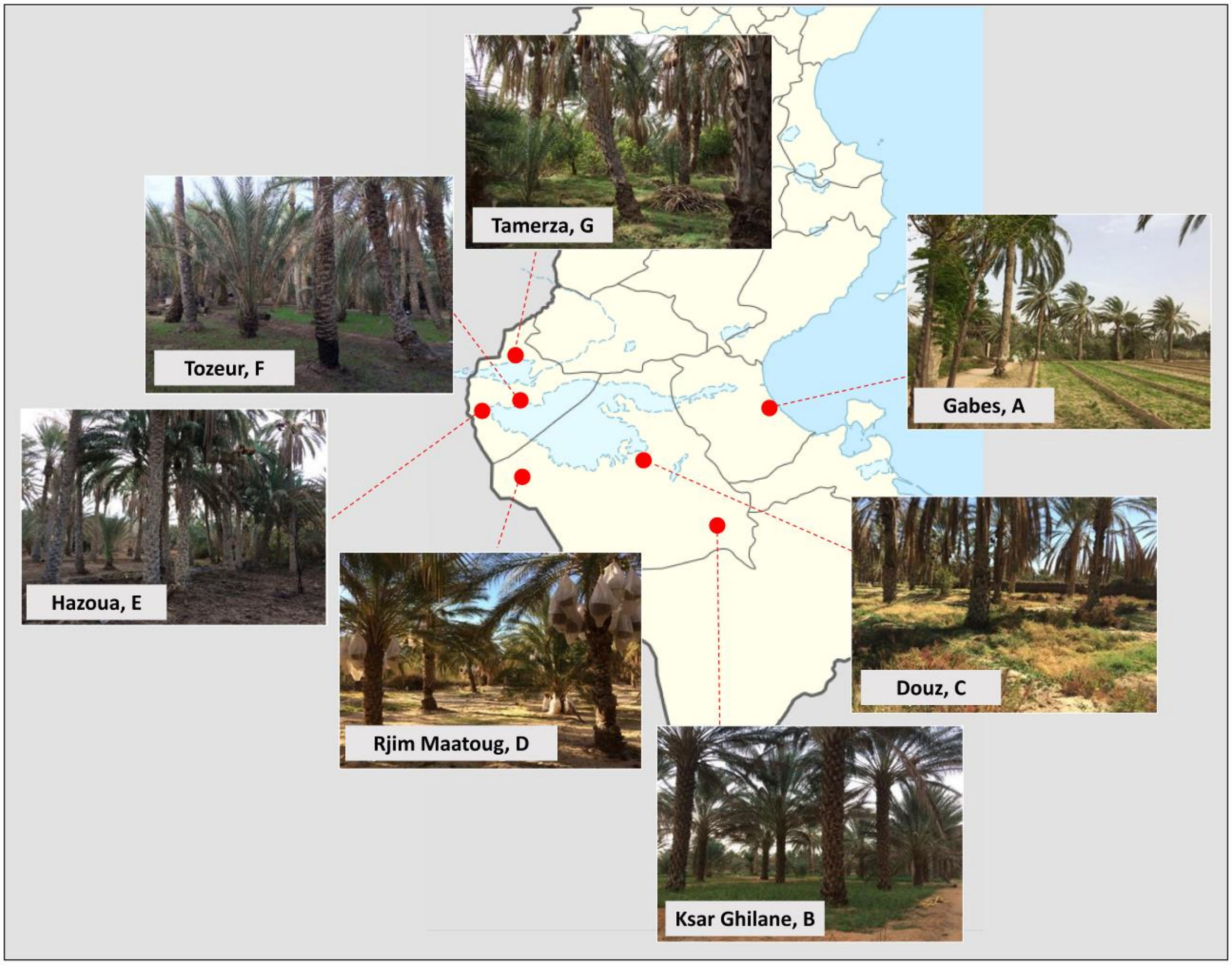

Location	Code	Classification	Coordinates	Influence	Climate
**Gabes**	A	Coastal	N36°19'27" E01°00'50"	Sea coast	Semi-arid
**Ksar Ghilane**	B	Saharan	N32°58'48" E09°38'19"	Desert	Saharan
**Douz**	C	Saharan	N33°26'75" E09°00'81"	Desert and Chott system	Saharan
**Rjim Maatoug**	D	Saharan	N33°31'80'' E07°98'82''	Desert	Saharan
**Hazoua**	E	Saharan	N33°73'81'' E07°59'97''	Desert and Chott system	Saharan
**Tozeur**	F	Saharan	N33°91'22'' E08°15'58''	Chott system	Saharan
**Tamerza**	G	Mountain	N34°23'00" E07°56'08"	Spring water and Chott system	Arid

Figure S2. Phylogenetic tree of PGP *Pseudomonas* spp. isolated from date palm root tissues (Cherif *et al*., 2015. *Env Microb Rep.* 7: 668-678) and *Pseudomonas* OTUs retrieved from the metagenomics analysis performed in this work.

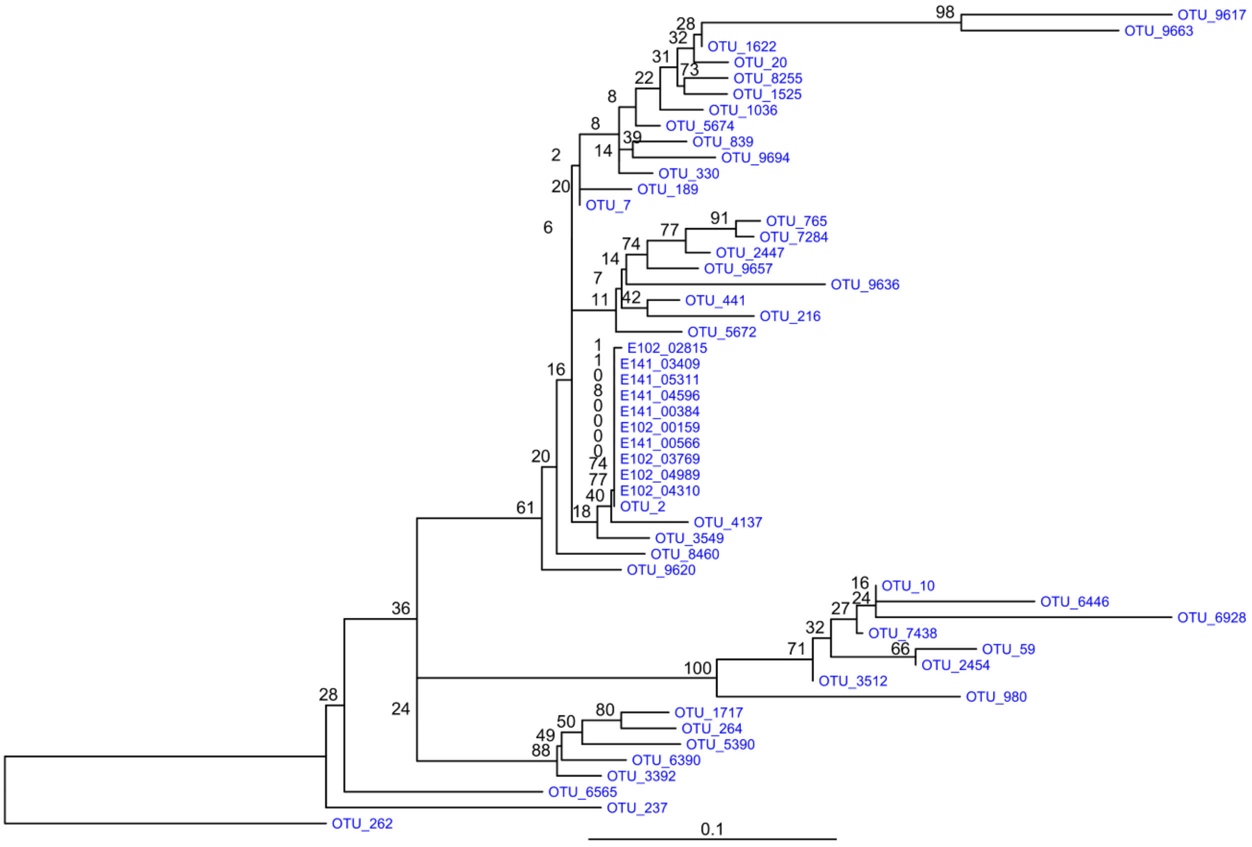

Figure S3. (**A**) Taxonomic affiliations of the nodes in the root, rhizosphere and bulk soil networks. (**B**) Intra- and inter-taxa bacterial interactions in the bacterial networks of core microbiota in each fraction. 'Edge betweenness' centrality values reveal the different distributions of node interactions at the phylum/class level for each fraction (bulk soil, rhizosphere and bulk soil).

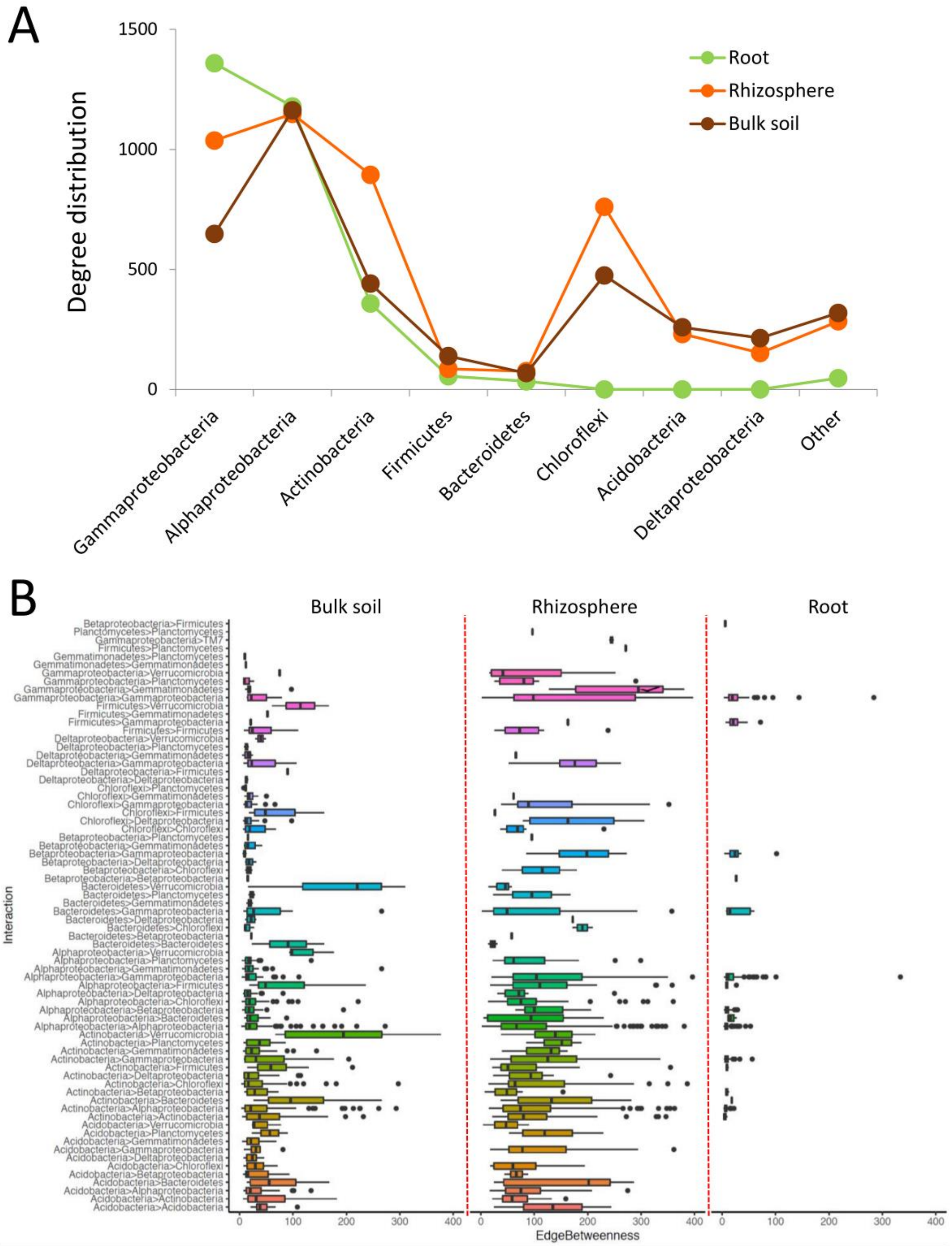

Figure S4. Centrality measure of network analysis. (**A**) Degree of connection, (**B**) average path length, (**C**) closeness centrality and (**D**) betweenness centrality are reported considering the taxonomic affiliation of the nodes in the three fractions (bulk soil, rhizosphere and root).

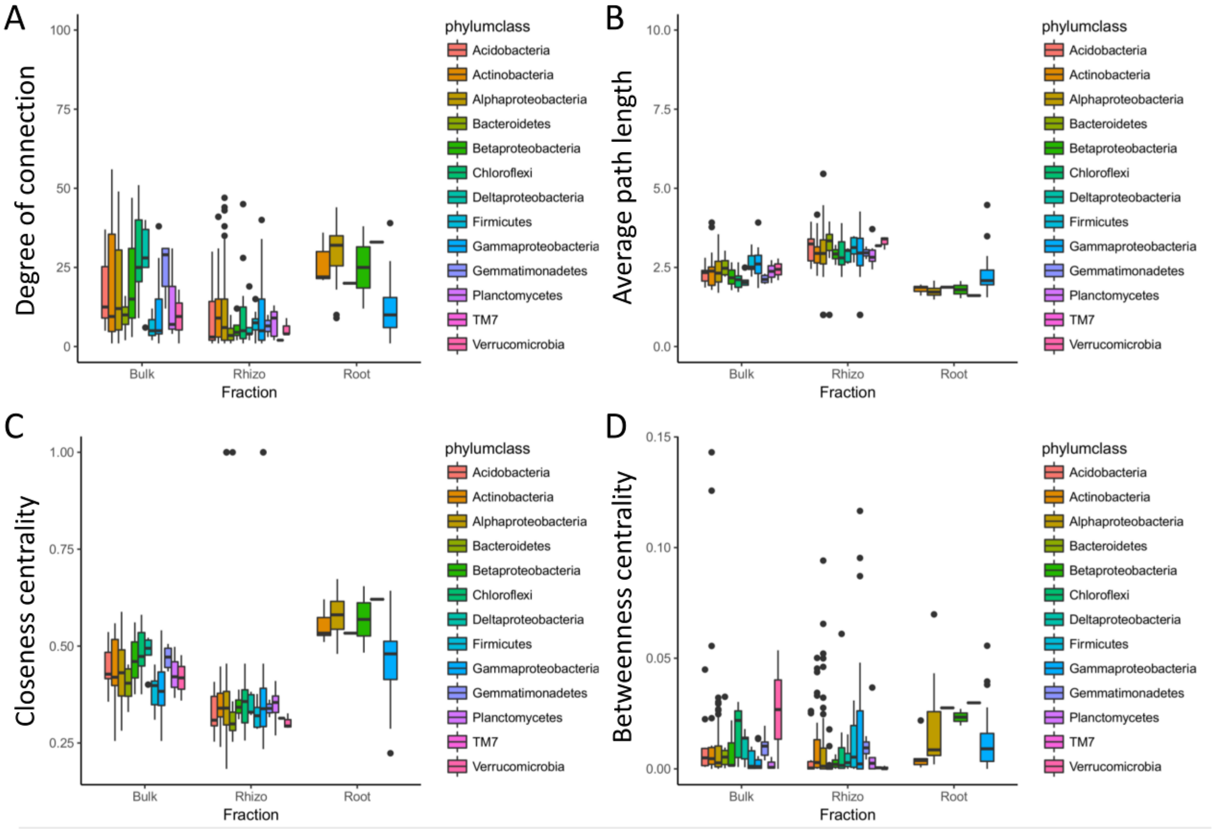

SUPPORTING TABLES

Table S1. Chemical analysis of root surrounding soil and bulk soil of the seven oases. Attached file Mosqueira et al 2018 SI Table S1.xlsx.

Table S2. Multiple comparisons among oasis soils. The seven oases are reported with capital letters: A = Gabes, B = Ksar Ghilane, C = Douz, D = Rejim Maatoug, E = Hazoua, F = Tozeur and G = Tamerza. Stars (*) indicate pairs of oases' soils that are significantly different.

**Oasis comparison**	**T**	**P(MC)**
A, B*	3.9104	0.001
A, C	1.3747	0.206
A, D*	4.8304	0.002
A, E*	5.4182	0.001
A, F*	3.1711	0.007
A, G*	4.3029	0.005
B, C	1.818	0.071
B, D*	2.3303	0.034
B, E*	2.1326	0.025
B, F*	2.6456	0.014
B, G*	1.8438	0.038
C, D	1.8871	0.068
C, E	1.8671	0.081
C, F*	1.8507	0.049
C, G	1.7704	0.096
D, E	1.6682	0.105
D, F*	3.2139	0.008
D, G*	2.169	0.037
E, F*	2.7382	0.018
E, G	1.323	0.232
F, G	1.8801	0.078

Table S3. Number of shared OTUs among the three fractions in each oasis studied.

A)

Fraction	N. of OTUs
Bulk	337
Rhizosphere	778
Root	267
Overall unique OTUs	867
**Core-Shared OTUs**	**101 (12%)**

B)

Fraction	N. of OTUs
Bulk	1035
Rhizosphere	717
Root	271
Overall unique OTUs	1090
**Core-Shared OTUs**	**213 (20%)**

C)

Fraction	N. of OTUs
Bulk	412
Rhizosphere	539
Root	279
Overall unique OTUs	731
**Core-Shared OTUs**	**104 (14%)**

D)

Fraction	N. of OTUs
Bulk	904
Rhizosphere	702
Root	309
Overall unique OTUs	1010
**Core-Shared OTUs**	**235 (23%)**

E)

Fraction	N. of OTUs
Bulk	1030
Rhizosphere	675
Root	160
Overall unique OTUs	1085
**Core-Shared OTUs**	**112 (10%)**

F)

Fraction	N. of OTUs
Bulk	1086
Rhizosphere	931
Root	358
Overall unique OTUs	1122
**Core-Shared OTUs**	**318 (28%)**

G)

Fraction	N. of OTUs
Bulk	1051
Rhizosphere	927
Root	206
Overall unique OTUs	1114
**Core-Shared OTUs**	**160 (14%)**

Table S4. Multi variate analysis of deviance to evaluate the percentage of bacterial community variance explained by (**A**) the interaction among location and fraction factors and (**B**) the one explained by the singles factors.

**A) Model Tested**	**Df**	**AIC**	**Difference AIC**	**%**
		202325		
Removing Fraction × Location	15012	227440	25115	12

**B) Model Tested**	**Df**	**AIC**	**Difference AIC**	**%**
		227440		
Removing Fraction	2502	275494	48054	21
Removing Location	7506	264059	36619	16

Table S5. Number of sequence and diversity indices calculated on the OTUs (97%) table considering (**A**) the factor 'Fraction' (root, rhizosphere and bulk soil) and (**B**) the interaction among factors 'Fraction' and 'Location'. Data have been reported as average ± standard error calculated on the 35 replicates and 5 replicates in (**A**) and (**B**), respectively. Results of one-way ANOVA (Tukey's Multiple Comparison Test) have been reported for Richness and Evenness diversity indices, considering the factor 'Fraction' or 'Location' (**A** and **B**, respectively). The seven oases are reported with capital letters: A = Gabes, B = Ksar Ghilane, C = Douz, D = Rejim Maatoug, E = Hazoua, F = Tozeur and G = Tamerza.

**(A) Fraction**	**N. sequence**	**Richness (N. OTUs)**	**Evenness (J)**
Root	37182 ± 3772	112 ± 10 (a)	0.379 ± 0.0277 (a)
Rhizosphere	19280 ± 1109	391 ± 36 (ab)	0.4936 ± 0.0283 (a)
Bulk soil	22056 ± 2865	615 ± 132 (b)	0.7657 ± 0.0433 (b)
ANOVA		***p* = 0.0011**	***p* < 0.0001**

**(B) Fraction**	**Location**	**N. sequence**	**Richness (N. OTUs)**	**Evenness (J)**
Root	A	29855 ± 7616	136 ± 19	0.4327 ± 0.0228
	B	44308 ± 10252	105 ± 21	0.3364 ± 0.0229
	C	20194 ± 4700	123 ± 21	0.3825 ± 0.0394
	D	41354 ± 9072	112 ± 34	0.2376 ± 0.1062
	E	45083 ± 4998	69 ± 6	0.4075 ± 0.0253
	F	47209 ± 4747	147 ± 37	0.3984 ± 0.0361
	G	32269 ± 3962	89 ± 10	0.458 ± 0.0159
ANOVA			*p* =0.29	*p* =0.06
Rhizosphere	A	20600 ± 9309	369 ± 105	0.5212 ± 0.0446
	B	13297 ± 2765	361 ± 70	0.5007 ± 0.0385
	C	22846 ± 3335	270 ± 36	0.3869 ± 0.0244
	D	18667 ± 2670	355 ± 59	0.3937 ± 0.0788
	E	19903 ± 4047	521 ± 58	0.5678 ± 0.0407
	F	19903 ± 5146	521 ± 115	0.5678 ± 0.0696
	G	19742 ± 6198	340 ± 138	0.5171 ± 0.066
ANOVA			*p* =0.64	*p* =0.20
Bulk soil	A	10366 ± 3891	95 ± 43 (a)	0.5867 ± 0.0611 (a)
	B	25017 ± 5633	737 ± 107 (bc)	0.7721 ± 0.0753 (bc)
	C	12075 ± 3668	158 ± 71 (a)	0.6227 ± 0.0622 (ac)
	D	24700 ± 6332	622 ± 55 (c)	0.8133 ± 0.0187 (b)
	E	26362 ± 3276	906 ± 31 (b)	0.8581 ± 0.0124 (b)
	F	26362 ± 3021	906 ± 18 (b)	0.8581 ± 0.0122 (b)
	G	29510 ± 5537	881 ± 30 (b)	0.8493 ± 0.0088 (b)
ANOVA			***p* < 0.0001**	***p* < 0.0001**

Table S6. Taxonomic composition of (**A**) total OTUs and (**B**) fraction-core microbiome. The seven oases are reported with capital letters: A = Gabes, B = Ksar Ghilane, C = Douz, D = Rejim Maatoug, E = Hazoua, F = Tozeur and G = Tamerza. Attached file Mosqueira et al 2018 SI Table S6.xlsx.
